# Reproductive history and cardiometabolic disease: the role of endogenous estrogen exposure across the lifespan in postmenopausal women

**DOI:** 10.1186/s12905-025-04030-5

**Published:** 2025-10-21

**Authors:** Yingze Zhu, Jialu Li, Wenjing Qin, Liang Wang, Qi Qi, Shaoru Li, Yue Cheng, Yuan Shen, Wenfang Yang, Zhonghai Zhu, Lingxia Zeng

**Affiliations:** 1 Department of Epidemiology and Biostatistics, School of Public Health, Xi’an Jiaotong University Health Science Center, Xi’an, 710061 Shaanxi China; 2 Heilongjiang Feihe Dairy Co., Ltd. Feihe Research Institute, Beijing, China; 3 Experimental Teaching Center, School of Public Health, Xi’an Jiaotong University Health Science Center, Xi’an, China; 4 Department of Nutrition and Food Safety Research, School of Public Health, Xi’an Jiaotong University Health Science Center, Xi’an, China; 5 Department of Obstetrics and Gynecology, Maternal and Child Health Center, The First Affiliated Hospital of Xi’an Jiaotong University, Xi’an, China; 6 Center for Chronic Disease Control and Prevention, Global Health Institution, Xi’an Jiaotong University, Xi’an, China; 7 Key Laboratory for Disease Prevention and Control and Health Promotion of Shaanxi Province, Xi’an, China

**Keywords:** Estrogen exposure, Reproductive history, Cardiometabolic disease, Cardiovascular diseases, Diabetes, Hypertension

## Abstract

**Background:**

The association between cumulative endogenous estrogen exposure across the lifespan, especially considering reproductive events, and women’s cardiometabolic health remain unclear. We aimed to examine the associations between lifetime endogenous estrogen exposure and the risks of diabetes, hypertension, and cardiovascular disease (CVD) among postmenopausal women.

**Methods:**

We used baseline data from the Regional Ethnic Cohort Study in Northwest China. Reproductive factors were self-reported using a structured questionnaire, and surrogate indicators of estrogen exposures—reproductive lifespan, endogenous estrogen exposure, cumulative gestation duration and other proportional indicators—were calculated. Diabetes, hypertension and CVD were defined based on self-reported physician diagnoses at the hospital. Multivariable logistic regression models were employed to estimate adjusted odds rations (aORs) and 95% confidence intervals (CIs).

**Results:**

Among 35,498 postmenopausal women (median age 59.0 years [interquartile range: 54.0–65.0], each additional year of reproductive lifespan was associated with lower risks of diabetes (aOR 0.971; 95%CI 0.961, 0.982), hypertension (aOR 0.969; 95%CI 0.962, 0.975) and CVD (aOR 0.954; 95%CI 0.946, 0.962). Similar inverse associations were observed for endogenous estrogen exposure. In contrast, a higher ratio of gestation-to-reproductive lifespan duration was positively associated with increased risks of diabetes, hypertension, and CVD. Multiple incomplete pregnancies were associated with increased diabetes risk, while multiple complete pregnancies were linked to elevated risks of hypertension and CVD.

**Conclusions:**

Longer exposure to endogenous estrogen was associated with decreased risk of cardiometabolic disease, while a higher burden of gestational events was associated with increased risks. Reproductive history could be considered as an indicator for risk stratification and management of cardiometabolic disease in women.

**Supplementary Information:**

The online version contains supplementary material available at 10.1186/s12905-025-04030-5.

## Introduction

Cardiovascular disease (CVD) remains the leading causes of disability and death worldwide [1]. As China experiences population growth and aging, CVD events are estimated to increase by over 50% by 2030 [2]. The rising average age of women is expected to further elevate the burden of CVD [3]. Moreover, the cardiovascular risk profile in women is distinct from that in men [4, 5]. For example, women are more likely to suffer cardiac rupture and fatal complications following a myocardial infarction [6], highlighting the need for sex-specific prevention and management strategies. Therefore, identifying CVD risk factors and implementing targeted screening and interventions for middle-to-older-aged women is crucial.

Among various women-specific factors, reproductive factors such as age at menarche, parity, and age at menopause have been increasingly linked to women’s vascular health [7]. A meta-analysis suggested that a longer reproductive lifespan is associated with a reduced risk of CVD [8], possibly due to the cardioprotective effect of endogenous estrogen [9]. Reproductive lifespan, defined as the interval between age at menarche and age at menopause, serves as the most widely used proxy of lifetime endogenous estrogen exposure [8]. Recent studies have further expanded the measure of lifetime endogenous estrogen exposure by incorporating additional reproductive events such as pregnancy and lactation to better capture lifetime endogenous exposure [10, 11].

However, there are inconsistencies in both the methods used to quantify lifetime endogenous estrogen exposure, and the observed associations between different endogenous estrogen exposure indicators and the risk of CVD [10, 12]. In addition, evidence on the associations between lifetime endogenous estrogen exposure and the risks of diabetes and hypertension—two major contributors to global disease burden involving vasculopathy—remains limited and yielded conflicting results [13].

In this study, we used baseline data from a large multi-ethnic cohort study in China to examine the associations between lifetime endogenous estrogen exposure and the risks of diabetes, hypertension and CVD. Specifically, we aim to (1) assess whether endogenous estrogen exposure is associated with cardiometabolic outcomes in a pattern similar to reproductive lifespan; and (2) quantify and specify the length and types of gestation across the reproductive lifespan to examine their associations with the cardiometabolic risks.

## Methods

### Study design and participants

This cross-sectional analysis used baseline data from postmenopausal women enrolled in the Regional Ethnic Cohort Study, an ongoing prospective cohort study among residents of five Northwest provinces in China. Participants were recruited between June 2018 and May 2019. Physical measurements and extensive questionnaires covering sociodemographic factors, lifestyle, medical history, and reproductive factors were administered by well-trained technicians at local clinical centers at enrollment. Detailed descriptions of the study design and recruitment have been published elsewhere [14].

### Exposures variables

Reproductive factors were collected at enrollment via face-to-face interviews using a structured questionnaire. Reproductive Lifespan was computed as age at menopause minus age at menarche. The number of complete pregnancies was derived from the sum of live births and stillbirths, while the number of incomplete pregnancies from the sum of spontaneous abortion, artificial abortion, and medical abortion. Lifetime cumulative gestational duration was estimated as 9 months for each complete pregnancy and 3 months for each incomplete pregnancy based on the average gestation lengths and previous studies [15, 16]. Considering the opposing effect of progesterone on estrogen during gestation [17] and the lower level of endogenous estrogen during lactation [18], endogenous estrogen exposure was calculated by subtracting the lifetime cumulative gestation and lactation duration from the reproductive lifespan.

Primary estrogen exposure indicators (in year) are defined as follows (see Supplemental Table 1):

$$\begin{aligned} &\mathrm{Reproductive}\;\mathrm{life}\;\mathrm{span}\\&=(\mathrm{age}\;\mathrm{at}\;\mathrm{menopause})-(\mathrm{age}\;\mathrm{at}\;\mathrm{menarche}) \end{aligned}$$

$$\begin{aligned} &\mathrm{Lifetime}\;\mathrm{cumulative}\;\mathrm{gestation}\;\mathrm{duration}\\&=(\mathrm{number}\;\mathrm{of}\;\mathrm{live}\;\mathrm{births}+\mathrm{number}\;\mathrm{of}\;\mathrm{still}\;\mathrm{births})\\&\times(9/12)+\mathrm{number}\;\mathrm{of}\;\mathrm{miscarriages}\times(3/12) \end{aligned}$$

$$\begin{aligned} &\mathrm{Endogenous}\;\mathrm{estrogen}\;\mathrm{exposure}\\&=(\mathrm{reproductive}\;\mathrm{lifespan})\\&-(\mathrm{lifetime}\;\mathrm{cumulative}\;\mathrm{gestation}\;\mathrm{duration})\\&-(\mathrm{lifetime}\;\mathrm{cumulative}\;\mathrm{lactation}\;\mathrm{duration}) \end{aligned}$$

To further analyze the relative contribution of each exposure indicator, we defined secondary exposure indicators as follows:

$$\begin{aligned} &\mathrm{Lifetime}\;\mathrm{complete}\;\mathrm{pregnancy}\;\mathrm{duration}\\&=(\mathrm{number}\;\mathrm{of}\;\mathrm{live}\;\mathrm{births}+\mathrm{number}\;\mathrm{of}\;\mathrm{still}\;\mathrm{births})\\&\times(9/12) \end{aligned}$$

$$\begin{aligned} &\mathrm{Lifetime}\;\mathrm{incomplete}\;\mathrm{pregnancy}\;\mathrm{duration}&\\&=\mathrm{number}\;\mathrm{of}\;\mathrm{miscarriages}\;\times\;(3/12) \end{aligned}$$

$$\begin{aligned} &\mathrm{Gestation}-\mathrm{to}-\mathrm{reproductive}\;\mathrm{lifespan}\;\mathrm{duration}\;\mathrm{ratio}\\&=(\mathrm{lifetime}\;\mathrm{cumulative}\;\mathrm{gestation}\;\mathrm{duration}/\\&\mathrm{reproductive}\;\mathrm{lifespan})\times100\% \end{aligned}$$

$$\begin{aligned} &\mathrm{Incomplete}\;\;\mathrm{pregnancy}-\mathrm{to}-\mathrm{lifetime}\;\mathrm{cumulative}\;\\&\mathrm{gestation}\;\mathrm{duration}\;\mathrm{ratio}=(\mathrm{lifetime}\;\mathrm{gestation}\;\mathrm{duration}\;\\&\mathrm{due}\;\mathrm{to}\;\mathrm{miscarriage}/\mathrm{lifetime}\;\mathrm{cumulative}\;\\&\mathrm{gestation}\;\mathrm{duration})\times100\% \end{aligned}$$

### Outcomes

Primary outcomes included diabetes, hypertension and CVD. Participants with a history of angina, other ischemic heart disease (IHD), acute myocardial infarction (AMI), or stroke were classified as having CVD. Secondary outcomes included angina, other IHD, AMI, and stroke. Data on these outcomes were collected through self-reported responses to the following question at enrollment: “Have you ever been diagnosed with diabetes by a doctor in a hospital at or above the county level?” Similar questions were used to collect information on hypertension, angina, other IHD, AMI, and stroke.

### Covariates

Covariates were selected based on prior literature and were collected at enrollment via face-to-face questionnaire and physical examination. In our study, we adjusted for a wide range of sociodemographic, lifestyle, reproductive and anthropometric factors, which may influence both reproductive history and cardiometabolic outcomes, to minimize potential confounding. Age was determined from identification card numbers. Ethnic groups were classified as Han or other minorities. Education levels were divided into five categories: no formal school, primary school, secondary school, high school, and college or above, following the Chinese education system and in line with prior studies using this cohort [14, 19]. Occupation types grouped into five categories: agriculture or factory worker, administrator or manager or technical, sales and service workers or self-employed, housewife or unemployed, and other or not stated. This categorization reflects typical socioeconomic positions in the study region and ensure adequate group sizes. Marital status was categorized into two groups: married and never married or separated or widowed or divorced. Household annual income was divided into five categories: <¥10,000, ¥10,000–49,999, ¥50,000–99,999, ≥¥100,000, and unclear. Tea, coffee, alcohol drinking, and smoking status were classified into two groups separately: never or almost never, and current. Physical activity was assessed using metabolic equivalents of task (MET) based on hours per day spent on occupation tasks, commuting, household tasks and sports activities, and then divided into quartiles. Body mass index (BMI) was calculated by dividing body weight in kilograms by the square of height in meters (kg/m^2^) and was further categorized as thinness (< 18.5 kg/m^2^), normal weight (18.5–23.9 kg/m^2^), overweight (24.0–27.9 kg/m^2^), and obesity (≥ 28.0 kg/m^2^) [20]. Age at menarche and age at first born were treated as continuous variables. Oral contraceptive (OCP) use history was categorized into three groups based on historical formulation changes: high dose group (used before 1975), low dose group (used after 1975), and never used [21]. Estrogen replacement therapy (ERT) history was binary (yes or no). Percentages of missing values in covariates were less than 1%, except for physical activity (8.68%) and age at first born (4.79%). Multiple imputation (Stata command, “mi”) via chained equations was used for the imputation of all missing values for 20 times.

### Statistical analysis

**Fig. 1:**
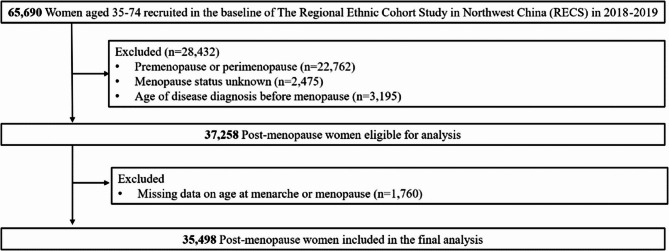
Flowchart

Figure 1 illustrates the data cleaning process. Women who were pre-menopause or perimenopause (*n* = 22,762), had missing menopause status (*n* = 2,475), or reported a diagnosis of outcomes before menopause (*n* = 3,195) were excluded. Additionally, cases with missing data on age at menarche or age at menopause were excluded (*n* = 1,760). The final sample size for analysis was 35,498.

Baseline characteristics were summarized as mean values with standard deviations (SD) for normally distributed continuous variables, and medians with interquartile ranges (IQR) for skewed continuous variables, and numbers with percentages for categorical variables. Associations of endogenous estrogen exposure with each outcome were assessed using logistic regression models, adjusted for sociodemographic characteristics (age at enrollment, province, ethnicity, educational level, occupation, marital status, household annual income), lifestyle factors (tea drinking, coffee drinking, alcohol consumption, smoking status, and physical activity in MET), reproductive factors (age at menarche, age at first born, OCP use history, and ERT history), and BMI. Multicollinearity between covariates was assessed using variance inflation factors (VIFs). Diabetes status was additionally adjusted when examining the association of endogenous estrogen exposure with hypertension and CVD. To explore associations between exposure and outcome, continuous exposure variables including reproductive lifespan, endogenous estrogen exposure and lifetime cumulative gestation duration were categorized into quartiles and treated as categorical variables in the regression models. To further investigate potential non-linear associations, we additionally applied restricted cubic spline (RCS) models with three knots placed at the 10th, 50th, and 90th percentiles of the exposures. We further examined potential interaction effects by sociodemographic factors and then conducted accordingly stratification analysis.

 In sensitivity analyses, we conducted similar analysis by: (1) excluding women with the history of hysterectomy, ovariotomy, lumpectomy, or cancer (*n* = 2,564), as these conditions may substantially alter reproductive lifespan and endogenous estrogen exposure, leading to potential misclassification of the exposure; (2) excluding women who had missing data on covariates (*n* = 5,498), to evaluate whether our results were affected by the handling of missing values. These two approaches aimed to confirm the robustness of the associations observed in the main analysis. In addition, as the prevalence of hypertension is nearly 20%, we also applied Poisson regression with robust variance to estimate risk ratios for hypertension [22]. Further, we applied E-values to assess the minimum strength of association that an unmeasured confounder would need to have with both the exposure and outcome to explain away observed associations [23]. A 2-sided *P* value less than 0.05 was considered statistical significance. All statistical analyses were conducted using Stata 15.0 (Stata Corp, College Station, Texas, USA).

## Results

A total of 35,498 post-menopause women with a median age of 59.0 years (IQR: 54.0–65.0) were enrolled in this study. Descriptive characteristics are summarized in Table 1. The median age at menarche and menopause of participants were 15.0 years (IQR: 14.0–17.0) and 49 years (IQR: 46.0–51.0), respectively, resulting in a median reproductive lifespan of 33.0 years (IQR: 30.0–36.0). After accounting for gestation and lactation durations, the median endogenous estrogen exposure was 28.50 years (IQR: 24.25–31.75). The proportions of diabetes, hypertension, and CVD were 5.41%, 19.72%, and 9.27%, respectively. The detailed information about disease status across subgroups of endogenous estrogen exposure were presented in the Supplemental Table 2.

**Table 1:** Baseline characteristics of postmenopausal participants (*N* = 35,498)

Variables	Descriptors
*Sociodemographic characteristics*	
Age at enrollment, median (IQR), y	59.00 (54.00–65.00)
Ethnicity, n (%)	
Han	27,464 (77.59)
Other minorities^a^	7,933 (22.41)
Province, n (%)	
Shaanxi	13,418 (37.80)
Xinjiang	9,817 (27.66)
Ningxia	5,476 (15.43)
Gansu	5,738 (16.16)
Qinghai	1,049 (2.96)
Education level, n (%)	
No formal school	12,032 (33.94)
Primary school	11,479 (32.38)
Junior high school	7,776 (21.94)
Senior high school	3,046 (8.59)
College and above	1,114 (3.14)
Occupation, n (%)	
Agriculture or factory worker	19,650 (55.77)
Administrator or manager or technical	1,292 (3.67)
Sales and service workers or self-employed	607 (1.72)
Housewife or unemployed	11,869 (33.68)
Other or not stated	1,819 (5.16)
Marital status, n (%)	
Married	30,400 (85.95)
Never married or separated or widowed or divorced	4,969 (14.05)
Household annual income, n (%)	
<¥10,000	7,891 (22.37)
¥10,000–49,999	19,857 (56.29)
¥50,000–99,999	5,170 (14.66)
≥¥100,000	1,007 (2.85)
Unclear	1,351 (3.83)
Tea drinking, n (%)	
Never or almost never	28,576 (80.50)
Now	6,992 (19.50)
Coffee drinking, n (%)	
Never or almost never	35,176 (99.09)
Now	322 (0.91)
Alcohol consumption, n (%)	
Never or almost never	28,576 (80.50)
Now	6,992 (19.50)
Smoking status, n (%)	
Never or almost never	34,830 (98.12)
Now	668 (1.88)
Physical activity in MET, median (IQR), h/d	20.10 (8.40–36.60)
BMI (kg/m^2^), n (%)	
< 18.5	1,154 (3.28)
18.5–23.9	15,217 (43.31)
24-27.9	13,215 (37.61)
≥ 28	5,552 (15.80)
*Reproductive characteristics*	
Age at menarche, median (IQR), y	15.00 (14.00–17.00)
Pregnancy events, median (IQR)	
Number of gravidities	3.00 (2.00–4.00)
Number of live births	2.00 (2.00–3.00)
Number of stillbirths	0.00 (0.00–0.00)
Number of incomplete pregnancies	0.00 (0.00–1.00)
History of oral contraceptive pills use, n (%)	
Never used	33,052 (93.11)
Low dose group	2,226 (6.27)
High dose group	220 (0.62)
History of estrogen replacement therapy, n (%)	
Never	35,284 (99.40)
Yes	214 (0.60)
Age at menopause, median (IQR), y	49.00 (46.00–51.00)
Endogenous estrogen exposures, median (IQR), y	
Reproductive life span	33.00 (30.00–36.00)
Lifetime lactation duration	2.50 (1.50-4.00)
Endogenous estrogen exposure	28.50 (24.25–31.75)
Lifetime cumulative gestational duration	1.75 (1.50–2.50)
Lifetime complete pregnancy duration	1.50 (1.50–2.25)
Lifetime incomplete pregnancy duration	0.00 (0.00-0.25)
*Major adverse vascular events*	
Cases of diabetes, n (%)	1,919 (5.41)
Cases of hypertension, n (%)	6,999 (19.72)
Cases of CVD, n (%)	3,290 (9.27)
Cases of angina, n (%)	911 (2.57)
Cases of other ischemic heart disease, n (%)	985 (2.77)
Cases of acute myocardial infarction, n (%)	592 (1.67)
Cases of stroke, n (%)	1,098 (3.09)

*Abbreviations* *IQR*, interquartile range *MET*, metabolic equivalents of task *CVD*, cardiovascular disease

^a^Other minorities refers to remaining 55 smaller ethnicities except for Han in China

**Fig. 2:**
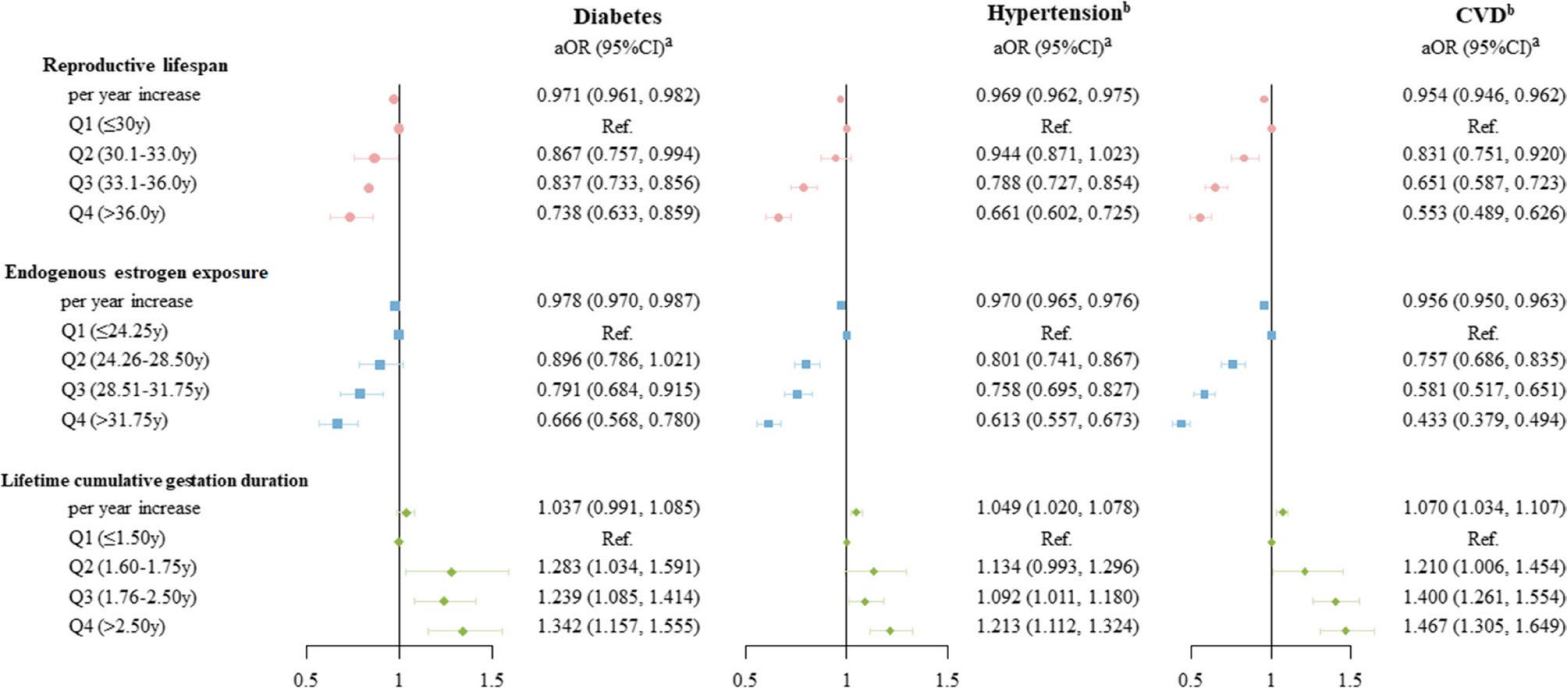
Associations between lifetime endogenous estrogen exposure and the risk of diabetes, hypertension, and CVD (*N* = 35,498) Abbreviations: CVD, cardiovascular disease. ^a^Adjustments include age at enrollment, province, ethnicity, educational level, occupation, marital status, household annual income, tea drinking, coffee drinking, alcohol consumption, smoke status, physical activity in MET, age at menarche, OCP use, age at first born, history of ERT, and BMI. ^b^Additionally adjusted diabetes in models

As shown in Fig. 2, each additional year of reproductive lifespan was associated with reduced risks of diabetes (adjusted odds ratio [aOR] 0.971; 95% confidence interval [CI] 0.961, 0.982), hypertension (aOR 0.969; 95%CI 0.962, 0.975) and CVD (aOR 0.954; 95%CI 0.946, 0.962). Compared with women in the lowest quartile, women in higher quartiles of reproductive lifespan had progressively lower risks of all three outcomes, showing inverse dose-response trends. Consistent associations were observed for endogenous estrogen exposure. Conversely, longer duration of lifetime cumulative gestation was positively associated with the risks of diabetes, hypertension and CVD, though the point estimate for diabetes was not statistically significant. Compared with women in the lowest quartile of lifetime cumulative gestation duration (≤ 1.5 years), those in the highest quartile (> 2.5 year) showed the most pronounced risks of diabetes (aOR 1.342; 95%CI 1.157, 1.555), hypertension (aOR 1.213; 95%CI 1.112, 1.324) and CVD (aOR 1.467; 95%CI 1.305, 1.649). Additionally, as shown in Supplemental Fig. 1, RCS models did not provide evidence for significant non-linear associations. The spline plots showed generally linear trends without clear “J” or “U” shape patterns. All VIF values for covariables were below 2.0, indicating low risk of collinearity and supporting the stability of model estimates.

As shown in Table 2, the ratio of gestation-to-reproductive lifespan duration was positively associated with the risks of diabetes (aOR: 1.019; 95%CI 1.007, 1.032), hypertension (aOR: 1.024; 95%CI 1.016, 1.032), and CVD (aOR:1.036; 95%CI 1.026, 1.045). However, the incomplete pregnancy-to-cumulative gestation duration ratio only showed a positive association with the risk of diabetes (aOR: 1.008; 95%CI 1.004, 1.012). Additionally, longer duration of incomplete pregnancy was associated with a 37% higher risk of diabetes, while longer duration of complete pregnancy was associated with a 2.0% higher risk of hypertension and a 4.3% higher risk of CVD.

**Table 2:** Association between lifetime pregnancy and the risk of diabetes, hypertension, and CVD (*N* = 35,498)

Indicators	Diabetes^a^			Hypertension^b^			CVD^b^	
	OR (95%CI)	*P* value		OR (95%CI)	*P* value		OR (95%CI)	*P* value
Lifetime complete pregnancy duration/per year	1.020 (0.973, 1.069)	0.416		1.043 (1.014, 1.074)	0.004		1.050 (1.014, 1.088)	0.006
Lifetime incomplete pregnancy duration/per year	1.374 (1.141, 1.656)	0.001		1.091 (0.966, 1.232)	0.159		1.159 (0.999, 1.345)	0.052
Gestation-to-reproductive lifespan duration ratio	1.019 (1.007, 1.032)	0.003		1.024 (1.016, 1.032)	< 0.001		1.036 (1.026, 1.045)	< 0.001
Incomplete pregnancy-to-cumulative gestation duration ratio	1.008 (1.004, 1.012)	< 0.001		1.002 (0.999, 1.004)	0.276		1.002 (0.998, 1.005)	0.311

*Abbreviations* *CVD*, cardiovascular disease

^a^ Adjustments include age at enrollment, province, ethnicity, educational level, occupation, marital status, household annual income, tea drinking, coffee drinking, alcohol consumption, smoke status, physical activity in MET, age at menarche, OCP use, age at first born, history of ERT, and BMI

^b^ Additionally adjusted diabetes in models

**Fig. 3:**
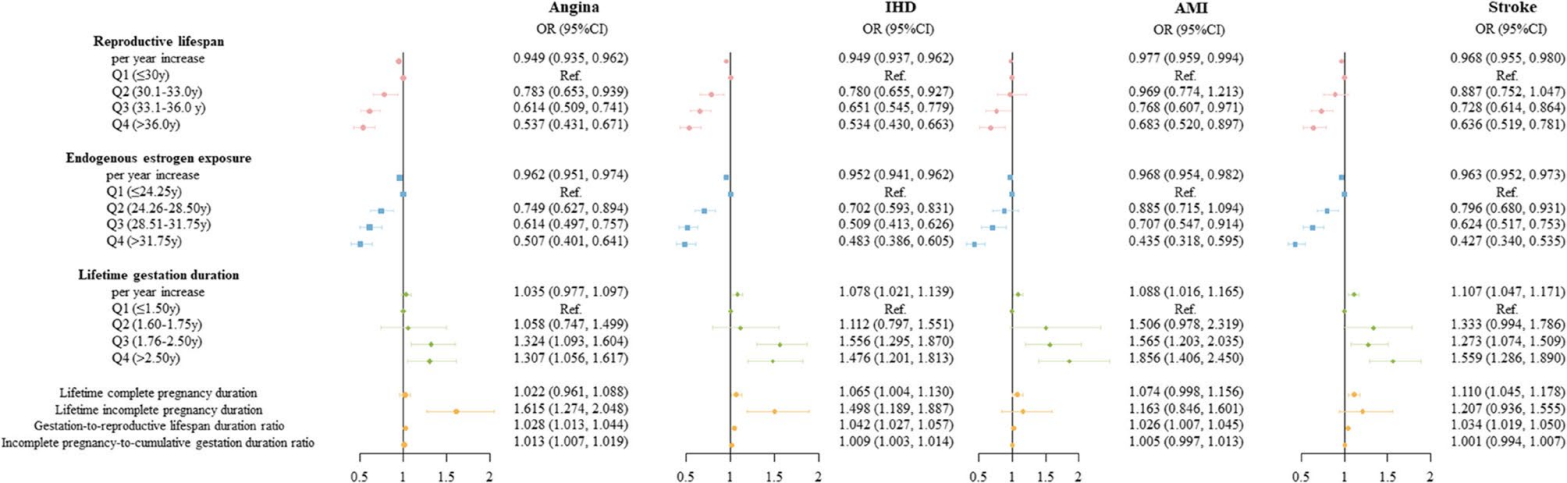
Associations of lifetime endogenous estrogen exposure and pregnancy experience with the risk of angina, other IHD, AMI, and stroke (*N* = 35,498) Abbreviations: IHD, ischemic heart disease; AMI, acute myocardialAbbreviations: IHD, ischemic heart disease; AMI, acute myocardial infarction. Adjustments include age at enrollment, province, ethnicity, educational level, occupation, marital status, household annual income, tea drinking, coffee drinking, alcohol consumption, smoke status, physical activity in MET, age at menarche, OCP use, age at first born, history of ERT, BMI, and diabetes

For secondary outcomes, analogous association patterns to those for CVD were observed (Fig. 3). Moreover, the associations of lifetime gestation due to complete pregnancy or incomplete pregnancy with the risks of secondary outcomes varied. Specifically, longer lifetime complete pregnancy duration was associated with higher risks of AMI and stroke, although the 95% CI for AMI included the null value (0.998–1.156; *P* = 0.055), while longer lifetime incomplete pregnancy duration was associated with a higher risk of angina. Notably, both longer lifetime complete pregnancy and incomplete pregnancy were associated with a higher risk of other IHD.

Significant interactions were observed for age and ethnicity. After stratified by age group (< (60 years old versus ≥ 60 years old) (Supplemental Table 3), longer reproductive lifespan and cumulative endogenous estrogen exposure were consistently associated with lower risks of diabetes, hypertension, and CVD across age groups, while the effect sizes were attenuated in women aged ≥ 60-years. For example, each additional year of reproductive lifespan was associated with a reduced risk of diabetes (< 60 years: aOR 0.944, 95% CI: 0.923, 0.965; ≥60 years: aOR 0.982, 95% CI: 0.969, 0.995). In contrast, longer lifetime gestation duration was associated with higher risks of diabetes and CVD among women aged ≥ 60 years. When stratified by ethnicity (Supplemental Table 4), consistent inverse associations of reproductive lifespan and cumulative estrogen exposure with diabetes, hypertension, and CVD were observed among both Han women and other minorities, with minor variations in effect sizes. However, prolonged lifetime gestation duration was associated with increased risk of diabetes only among Han women, and with increased risk of hypertension only among other minor ethnicities.

In sensitivity analysis, consistent results were found among subpopulations after excluding women with a history of hysterectomy, ovariotomy, lumpectomy, or cancer (see Supplemental Table 5) and women with missing data on covariates (see Supplemental Table 6), suggesting the robustness of our results. Sensitivity analyses using Poisson regression with robust variance estimation yielded results that were consistent with the main logistic regression findings, though with slightly attenuated effect size (Supplementary Table 7). The E-values for the effect estimates and their CIs were shown in the Supplemental Table 8. For example, the E-value for the point estimate and upper CI for the association between the highest quartile of reproductive lifespan and the risk of CVD were 3.017 and 2.574, respectively, indicating that an unmeasured confounder would need to be strongly associated with both the exposure and the outcome to completely negate the observed association.

## Discussion

In this study, we observed a consistent inverse association of the duration of reproductive lifespan and lifetime endogenous estrogen exposure with the risks of diabetes, hypertension and CVD among postmenopausal women in Northwest China. Women experienced longer cumulative gestation duration exhibited higher risks of diabetes, hypertension and CVD. In addition, multiple incomplete pregnancies were associated with higher risk of diabetes, while multiple complete pregnancies were associated with higher risks of hypertension and CVD. These findings highlight the value of incorporating women-specific reproductive history into risk stratification and cardiometabolic disease prevention strategies.

### Lifetime endogenous Estrogen exposure and diabetes

Our results demonstrated that a longer reproductive lifespan was associated with a decreased risk of diabetes among postmenopausal women. This result is consistent with a previous meta-analysis synthesized data from two cohort studies published in 2022, reporting a 6–15% decreased diabetes risk per additional year of reproductive lifespan [13]. However, few large-scale studies has provided evidence for the association between lifetime cumulative endogenous estrogen exposure with the risk of diabetes [13]. In our study, by incorporating the durations of gestation and lactation, which are key periods characterized by significant estrogen fluctuations to assess endogenous estrogen exposure, we observed similar inverse while slightly attenuated associations. This nuanced approach advances prior research by considering dynamic hormonal changes across reproductive lifespan, supporting the protective role of endogenous estrogen in glucose homeostasis and insulin sensitivity [24]. Similarly, a prospective cohort study conducted in Australia observed that a higher risk of incident diabetes at midlife was found among women experiencing a shorter reproductive lifespan [25]. However, observational studies conducted in China showed varying results, indicating no association or non-linear relationship between reproductive lifespan and the risk of diabetes [26, 27]. These discrepancies may stem from variations in sample size, study design, population age ranges, and adjustments for potential confounders. Moreover, diverse socioeconomic status and environmental factors among different populations may contribute to divergent estrogen metabolism and diabetes susceptibility profiles. Such variability highlights the critical need for harmonized, large-scale cohort studies with standardized protocols across diverse populations to clarify these complex relationships.

Additionally, a longer lifetime cumulative gestation duration was associated with an increased risk of diabetes. Notably, multiple incomplete pregnancies, rather than complete pregnancies were associated with higher risk of diabetes. When applied the derived indicators of gestation-to-reproductive lifespan duration ratio and incomplete pregnancy-to-cumulative gestation duration ratio, both of them showed positive associations with the risk of diabetes, highlighting the adverse impact of incomplete pregnancy on diabetes. However, the Women’s Health Initiative cohort in the United States, with an average follow-up of 14.2 years, found that a greater number of complete pregnancies was associated with increase risk of diabetes [28]. Another study conducted in China showed each additional pregnancy increases the risk of diabetes by 4%, and a history of pregnancy loss was associated with a 7% higher risk of diabetes [29]. Furthermore, recently a meta-analysis suggested that women underwent three or more miscarriages had a 1.99-fold risk of non-gestational diabetes (95%CI 1.36, 2.91) compared to those never experiencing a miscarriage [30]. The observed association between incomplete pregnancy and the risk of diabetes potentially due to the multiple pathways. First, abrupt hormonal fluctuations following pregnancy loss might play role [31, 32]. Rapid declines in estrogen and progesterone levels may disrupt metabolic homeostasis, leading to systemic inflammation and insulin resistance [33]. Second, incomplete pregnancies, particularly recurrent ones, may impose substantial psychological stress and emotional burden. Chronic stress can activate the hypothalamic–pituitary–adrenal axis and sympathetic nervous system, contributing to metabolic dysregulation [34, 35]. Third, multiple incomplete pregnancy may also reflect underlying health conditions such as metabolic syndrome, insulin resistance, polycystic ovary syndrome, which have been well-established risk factors for diabetes [36, 37]. In our study, in contrast to incomplete pregnancies, complete pregnancies were not associated with the risk of diabetes. The metabolic adaptations induced by pregnancy (e.g., transient insulin resistance) typically resolve postpartum, and the metabolic “reset” may be enhanced by lactation [38, 39]. In addition, for live births, lactation duration has been associated with improvements in glucose metabolism and reduced diabetes risk among women with prior gestational diabetes [40]. Further prospective research using longitudinal direct biomarker data is needed to unravel relationship and mechanisms through how the cumulative effect of endogenous estrogen exposure influences the risk of metabolic disease.

### Lifetime endogenous Estrogen exposure, hypertension and CVD

Our findings align with prior epidemiological studies conducted in non-Asian population suggesting a protective effect of lifetime cumulative estrogen exposure on vascular health [10, 12]. By confirming these associations in a multi-ethnic population in Northwestern China, our study extends this evidence to an underrepresented setting. A meta-analysis synthesized 17 studies and concluded that a shorter reproductive lifespan was associated with a higher risk of CVD events, particularly stroke (pooled relative risk: 1.31; 95%CI 1.25, 1.36) [8]. Endogenous estrogen exposure also yielded comparable results, suggesting the beneficial role of endogenous estrogen in promoting vasorelaxation, sympathoinhibition, preventing vascular remodeling, and providing renoprotection [41]. However, some inconsistencies remain. An analysis from the Australian Longitudinal Study on Women’s Health found inverse association between the length of reproductive lifespan, but this association disappeared when using a derive indicator of endogenous estrogen exposure indicator incorporated pregnancies, breastfeeding, miscarriage, and OCP use [10]. Another study, which defined further refined the estimation by differentiating estrogen-dominant and progesterone-dominant phases of the menstrual cycle, showed that shorter durations of exposure to endogenous estrogen was associated higher risk of CVD [12]. Except for study population, setting, and confounders included in models, these discrepancies might mainly due to the differences in how endogenous estrogen exposure is defined and calculated across studies. For example, the Australian study defined endogenous estrogen exposure by subtracting pregnancy (9 months/per child), breastfeeding (4 months/per child), miscarriages/terminations (3 months/per miscarriage/termination), and OCP (years) from reproductive lifespan [10]. While another mentioned study refined this by focusing only on estrogen-dominant (follicular) phases, excluding progesterone-dominant (luteal) phases, pregnancies (40 weeks per birth or 20 weeks per abortion), progestogen-containing contraceptive use, and breastfeeding [12]. Endogenous estrogen regulates metabolic markers, such as lipids, inflammatory markers, and coagulates in blood-vascular systems to show a cardioprotective effect [42]. Additionally, a shorter reproductive lifespan may reflect accelerated biological aging, which could exacerbate vascular damage and metabolic dysregulation in midlife. The rising risk of hypertension and CVD with a decreasing length of cumulative estrogen exposure may be due to both direct effect of hormonal changes on the vasculature and metabolic changes associated with ageing [43]. Therefore, these results highlight the need for standardized, harmonized methods to estimate lifetime estrogen exposure, and underscore the importance of considering detailed reproductive histories in cardiovascular risk assessment for women. In addition, significant interactions were observed for age and ethnicity in this study.

In our study, women in the highest cumulative gestation duration quartile faced a 21% increased risk of hypertension and a 47% elevated risk of CVD compared to the lowest group. Notably, we observed multiple complete pregnancies were associated with higher risks of hypertension, overall CVD, angina and IHD, while multiple incomplete pregnancies were associated with the increased risks of IHD, AMI and stroke. These varying association profiles may indicate different underlying pathological mechanism for specific CVD subtypes. However, data from China Kadoorie Biobank revealed that longer duration of pregnancy (both complete and incomplete) was associated with an increased risk of stroke [16]. Additionally, many of existing studies treated CVD as an overall outcome or only examined one of the CVD outcomes (such as coronary heart disease, stroke) [44, 45]. The associations between pregnancy type and specific CVD subtype remain unestablished and unclear. Our study provides a more nuanced perspective by distinguishing the differential associations of complete versus incomplete pregnancies on specific CVD outcomes.

For complete pregnancies, although previous studies reported inconsistent conclusions regarding the association between parity or gravidity and vascular health, with some showing a “J shape” relationship in which women with one or two children have the lowest CVD risk [46], and others indicating a linear increase in risk with each additional pregnancy [47], it is well established that pregnancy is characterized by profound physiological and metabolic changes across multiple organ systems [48]. Furthermore, Eunhee and Leslie have proposed pregnancy as a cardiac stress model, revealing an underlying predisposition to future cardiovascular disease in mothers [49]. Repeated complete pregnancies may have a cumulative and long-lasting impact on women’s vascular health through sustained physiological changes [50] and the psychological burden [51] experienced across the lifespan. Pregnancy induces profound physiological and metabolic adaptations, including transient insulin resistance, hemodynamic changes, and vascular remodeling, which typically resolve postpartum but may persist or accumulate after multiple pregnancies. Moreover, pregnancy complications such as hypertensive disorders and gestational diabetes, which are increasingly prevalent with delayed gestational weeks can further cumulative and amplify long-term cardiometabolic risk [52]. Although our study lacked detailed information on pregnancy complications and their contribution to later-life CVD, these factors should be addressed in future research. These factors warrant careful consideration in future research. In addition, pregnancy-induced changes, such as excess gestational weight gain and perinatal depression, have been linked to the increased CVD risk [53]. Therefore, repeated complete pregnancies could lead to a gradual accumulation of vascular stress, endothelial dysfunction, and metabolic strain, thereby elevating the risks of hypertension and CVD across the life course.

A large-scale international study synthesized data from eight prospective cohort studies observed positive association between recurrent pregnancy loss and the risk of non-fatal stroke [54]. Evidence from a meta-analysis have shown that a history of miscarriage or recurrent miscarriage is associated with a greater risk of coronary hearth disease [45]. Our study verified the associations between incomplete pregnancies and the risk of CVD in later life in a multi-ethnicity Chinese population. Incomplete pregnancies may contribute to cardiovascular risk through several biological pathways. Incomplete pregnancy may increase the risk of CVD through shared vascular pathology, such as systemic inflammation, elevated level of homocysteine, and endothelial dysfunction [45]. Besides, alloimmune and autoimmune factors, which play important roles in pregnancy loss, especially recurrent miscarriages, are linked to arterial thrombosis, including the presence of antiphospholipid antibodies [55]. Thus, multiple incomplete pregnancies might lead to the cumulation of vascular and endothelial dysfunction. Additionally, incomplete pregnancies may also serve as markers of underlying health conditions such as metabolic syndrome, insulin resistance, or polycystic ovary syndrome, which elevate cardiometabolic risk [36, 37]. Therefore, incomplete pregnancies may serve both as an independent vascular risk factor and as an indicator of pre-existing pathophysiological susceptibility. Taken together, while our findings suggest potential associations between pregnancy history and cardiometabolic outcomes, these results should be interpreted cautiously given the unmeasured confounders and observational nature of the study. Further research should disentangle these distinct pathways and further clarify pathophysiological mechanisms of specific CVD types.

### Strength and limitations

The large sample size and well characterized nature of participants allowed a detailed evaluation of the associations of endogenous estrogen exposures with diabetes, hypertension, and CVD. In addition to the reproductive lifespan, we accounted for fluctuations in endogenous estrogen levels resulting from other reproductive histories, including pregnancy and lactation. Gestation duration due to complete and incomplete pregnancy was assessed separately to determine if associations differ by pregnancy type. Furthermore, except for common confounding factors, we adjusted other key reproductive factors in models such as age at first birth, OCP use and ERT history. Additionally, our stratified analyses further suggest that the association between endogenous estrogen exposure and the risks of cardiometabolic weaken with advancing age, while prolonged gestational duration may exert adverse cumulative impacts later in life, suggesting the influence of biological aging. The observed ethnic heterogeneity indicates that genetic and environmental contexts may modify these associations. These analyses provide a better understanding of the association between estrogen exposure across reproductive lifespan and vascular health profile in later life.

Our findings should be interpreted with a few limitations. Firstly, the cross-sectional design does not allow causal inferences about the associations of estrogen exposure indicators with diabetes, hypertension and CVD. While, to provide a clear research background and discuss potential mechanisms, some terms used in the manuscript, such as “impact”, are intended to indicate associations but not causal link. Nevertheless, the consistency of our main results with those from prospective cohort studies supports the plausibility of the observed associations and provides a basis for future hypothesis-driven research. Secondly, self-reported reproductive factors may introduce recall bias. However, previous studies have demonstrated moderate to high agreement between adulthood recall of key reproductive milestones—such as age at menarche—and their actual timing, supporting the reliability of these self-reported measures [56]. Thirdly, surrogate indicators of endogenous estrogen exposures were constructed based on key reproductive history, which can not fully capture the real dynamic hormonal fluctuations across the lifespan. This may introduce measurement error and potential misclassifications. Although these proxies have been widely used in large-scale epidemiological research, further research using more rigorous design and direct hormonal measurements are warranted to validate estimates of lifetime estrogen exposure. Nevertheless, these key reproductive histories could serve as a simple and easy approach to stratify population with higher risks of cardiometabolic diseases and further screen and disease management. Fourthly, disease status was assessed based on self-reported physician diagnoses, which may lead to underestimation of cardiometabolic disease prevalence and potential misclassification, which could further attenuate the observed associations [57]. However, severe conditions of diabetes, hypertension, and CVD typically require medical treatment and ongoing management, making it less likely for patients to underreport. Furthermore, previous studies conducted in China have demonstrated substantial agreement between self-reported and biomedical measurement of hypertension and diabetes [58, 59], supporting the reasonable reliability of self-reported disease status in this context. In addition, to ensure the correct temporal sequence between reproductive history and cardiometabolic outcomes, we excluded women who reported CMD prior to menopause, which may have further reduced the observed prevalence in our final analytic sample. Lastly, despite adjusting for major confounders, unmeasured or residual confounding may still affect the observed associations, such as genetic factors, early life factors, complications during pregnancy, medications use as well as other socioeconomic variables. However, the calculated E-values indicate that substantial unmeasured confounder would be required to fully explain observed associations with the cardiometabolic disease.

## Conclusions

In summary, this study provides observational evidence suggesting that lifetime endogenous estrogen exposure was associated with women’s vascular and metabolic health. A longer duration of lifetime endogenous estrogen exposure is associated with decreased risks of diabetes, hypertension and CVD, whereas prolonged cumulative gestation duration, particular incomplete pregnancies was associated with increased risks. These findings highlight the value of integrating reproductive history into cardiovascular risk assessment tools and tailoring prevention strategies to address sex-specific risk factors. Future research should include diverse populations and adopt rigorous study designs to clarify the underlying biological mechanisms and develop targeted interventions to mitigate reproduction-related vascular risks.

## Supplementary Information

Supplementary Material 1

## Data Availability

All data are not publicly available due to ethical and privacy reasons, but can be obtained from the corresponding author upon reasonable request.

## Abbreviations

aOR: Adjusted Odds Ratio
AMI: Acute Myocardial Infarction
BMI: Body Mass Index
CI: Confidence Interval
CVD: Cardiovascular Disease
ERT: Estrogen Replacement Therapy
IHD: Ischemic Heart Disease
IQR: Interquartile Range
MET: Metabolic Equivalent of Task
OCP: Oral Contraceptive Pills
RCS: Restricted Cubic Spline
SD: Standard Deviation
VIF: Variation Inflation Factor
