# Supplementary material for: Reproductive history and cardiometabolic disease: the role of endogenous estrogen exposure across the lifespan in postmenopausal women
Source: BMC Womens Health. 2025 Oct 21;25:501. doi: 10.1186/s12905-025-04030-5 (PMC12538824; doi:10.1186/s12905-025-04030-5)

**Supplemental Table 1. Indicators assessing estrogen exposure across women’s lifespan in this study.**

| Indicators | Algorithm to calculate indicator |
| --- | --- |
| Reproductive lifespan | age at menopause − age at menarche |
| Lifetime cumulative gestation duration | (number of live births + number of stillbirth) × (9/12) + number of miscarriages × (3/12) |
| Lifetime cumulative lactation duration | the sum lactation duration for the live births |
| Endogenous estrogen exposure | reproductive lifespan − lifetime cumulative gestation duration – lifetime cumulative lactation duration |
| Lifetime complete pregnancy duration | (number of live births + number of still births) × (9/12) |
| Lifetime incomplete pregnancy duration | number of miscarriages × (3/12) |
| Gestation-to-reproductive lifespan duration ratio | (lifetime cumulative gestation duration / reproductive lifespan) × 100% |
| Incomplete pregnancy-to-cumulative gestation ratio | (lifetime gestation duration due to miscarriage / lifetime cumulative gestation duration) × 100% |

**Supplemental Table 2. Proportion of diabetes, hypertension, and CVD among subgroups of lifetime endogenous estrogen exposures.**

| Estrogen exposure indicators | Diabetes, n(%) | Hypertension, n(%) | CVD, n(%) |
| --- | --- | --- | --- |
| Reproductive lifespan/per year |  |  |  |
| Q1 (≤30y) | 583 (5.94) | 2194 (22.35) | 1228 (12.51) |
| Q2 (30.1-33.0y) | 420 (5.15) | 1635 (20.05) | 777 (9.53) |
| Q3 (33.1-36.0 y) | 540 (5.22) | 1893 (18.31) | 787 (7.61) |
| Q4 (>36.0y) | 376 (5.23) | 1277 (17.76) | 498 (6.93) |
| Endogenous estrogen exposure/per year |  |  |  |
| Q1 (≤24.25y) | 616 (6.79) | 2440 (26.89) | 1399 (15.42) |
| Q2(24.26-28.50y) | 505 (5.54) | 1732 (18.99) | 865 (9.48) |
| Q3(28.51-31.75y) | 396 (4.69) | 1440 (17.05) | 566 (6.70) |
| Q4 (>31.75y) | 402 (4.54) | 1387 (15.66) | 460 (5.19) |
| Lifetime cumulative gestation duration/per year |  |  |  |
| Q1 (≤1.50y) | 599 (3.72) | 2229 (13.85) | 889 (5.52) |
| Q2(1.60-1.75y) | 114 (5.52) | 356 (17.24) | 154 (7.46) |
| Q3(1.76-2.50y) | 526 (5.81) | 1825 (20.17) | 940 (10.39) |
| Q4(>2.50y) | 680 (8.20) | 2589 (31.23) | 1307 (15.77) |

Abbreviations: CVD, cardiovascular disease.

**Supplemental Table 3**. Association between endogenous estrogen exposure indicators and diabetes, hypertension, CVD after stratified by age group.

| Endogenous estrogen exposure indicators | Diabetes / aOR (95% CI) | |  | Hypertension / aOR (95% CI) | |  | CVD / aOR (95% CI) | |
| --- | --- | --- | --- | --- | --- | --- | --- | --- |
|  | <60 year-old | ≥ 60 year-old |  | <60 year-old | ≥ 60 year-old |  | <60 year-old | ≥ 60 year-old |
| Reproductive lifespan/per year | 0.944 (0.923, 0.965) | 0.982 (0.969, 0.995) |  | 0.925 (0.914, 0.936) | 0.984 (0.976, 0.992) |  | 0.921 (0.907, 0.935) | 0.967 (0.957, 0.977) |
| Endogenous estrogen exposure/per year | 0.950 (0.931, 0.969) | 0.986 (0.976, 0.997) |  | 0.935 (0.925, 0.944) | 0.983 (0.976, 0.990) |  | 0.936 (0.924, 0.949) | 0.936 (0.955, 0.971) |
| Lifetime gestation duration/per year | 1.058 (0.947, 1.183) | 1.076 (1.018, 1.137) |  | 1.085 (1.024, 1.149) | 1.059 (1.022, 1.098) |  | 1.053 (0.977, 1.134) | 1.122 (1.075, 1.171) |

Abbreviations: CVD, cardiovascular disease.

**Supplemental Table 4**. Association between endogenous estrogen exposure indicators and diabetes, hypertension, CVD after stratified by ethnicity

| Endogenous estrogen exposure indicators | Diabetes / aOR (95% CI) | |  | Hypertension / aOR (95% CI) | |  | CVD / aOR (95% CI) | |
| --- | --- | --- | --- | --- | --- | --- | --- | --- |
|  | Han | Other minorities |  | Han | Other minorities |  | Han | Other minorities |
| Reproductive lifespan/per year | 0.974 (0.961, 0.987) | 0.977 (0.957, 1.998) |  | 0.969 (0.961, 0.978) | 0.966 (0.954, 0.977) |  | 0.955 (0.945, 0.966) | 0.957 (0.944, 0.971) |
| Endogenous estrogen exposure/per year | 0.974 (0.963, 0.986) | 0.988 (0.972, 1.004) |  | 0.977 (0.970, 0.985) | 0.971 (0.962, 0.980) |  | 0.954 (0.945, 0.964) | 0.961 (0.951, 0.972) |
| Lifetime gestation duration/per year | 1.169 (1.084, 1.260) | 1.008 (0.939, 1.082) |  | 0.994 (0.946, 1.045) | 1.045 (1.003, 1.088) |  | 1.140 (1.067, 1.219) | 1.079 (1.029, 1.131) |

Abbreviations: CVD, cardiovascular disease.

**Supplemental Table 5.** **Sensitivity analyses among women without the history of hysterectomy, ovariotomy, lumpectomy, or cancer (N=32,934).**

| Estrogen exposure indicators | diabetes ^a^ | |  | hypertension ^b^ | |  | CVD ^b^ | |
| --- | --- | --- | --- | --- | --- | --- | --- | --- |
|  | OR (95%CI) | *P* value |  | OR (95%CI)^a^ | *P* value |  | OR (95%CI)^a^ | *P* value |
| Reproductive lifespan/per year | 0.976 (0.965, 0.988) | <0.001 |  | 0.972 (0.966, 0.979) | <0.001 |  | 0.955 (0.947, 0.963) | <0.001 |
| Q1 (≤30y) | Ref. |  |  | Ref. |  |  | Ref. |  |
| Q2 (30.1-33.0y) | 0.902 (0.782, 1.041) | 0.158 |  | 0.988 (0.908, 1.075) | 0.774 |  | 0.859 (0.773, 0.956) | 0.005 |
| Q3 (33.1-36.0 y) | 0.864 (0.751, 0.994) | 0.041 |  | 0.816 (0.750, 0.888) | <0.001 |  | 0.670 (0.600, 0.748) | <0.001 |
| Q4 (>36.0y) | 0.776 (0.661, 0.911) | 0.002 |  | 0.691 (0.627, 0.761) | <0.001 |  | 0.572 (0.502, 0.651) | <0.001 |
| Endogenous estrogen exposure/per year | 0.982 (0.973, 0.991) | <0.001 |  | 0.973 (0.968, 0.979) | <0.001 |  | 0.957 (0.950, 0.964) | <0.001 |
| Q1 (≤24.25y) | Ref. |  |  | Ref. |  |  | Ref. |  |
| Q2(24.26-28.50y) | 0.944 (0.823, 1.083) | 0.411 |  | 0.814 (0.749, 0.884) | <0.001 |  | 0.760 (0.686, 0.842) | <0.001 |
| Q3(28.51-31.75y) | 0.820 (0.703, 0.956) | 0.011 |  | 0.774 (0.707, 0.848) | <0.001 |  | 0.590 (0.523, 0.666) | <0.001 |
| Q4 (>31.75y) | 0.710 (0.601, 0.838) | <0.001 |  | 0.636 (0.576, 0.702) | <0.001 |  | 0.435 (0.379, 0.500) | <0.001 |
| Lifetime cumulative gestation duration/per year | 1.040 (0.992, 1.091) | 0.103 |  | 1.053 (1.023, 1.084) | <0.001 |  | 1.078 (1.040, 1.116) | <0.001 |
| Q1 (≤1.50y) | Ref. |  |  | Ref. |  |  | Ref. |  |
| Q2(1.60-1.75y) | 1.308 (1.043, 1.641) | 0.020 |  | 1.131 (0.983, 1.301) | 0.087 |  | 1.140 (0.934, 1.391) | 0.197 |
| Q3(1.76-2.50y) | 1.263 (1.100, 1.450) | 0.001 |  | 1.095 (1.010, 1.187) | 0.027 |  | 1.385 (1.241, 1.545) | <0.001 |
| Q4(>2.50y) | 1.328 (1.138, 1.550) | <0.001 |  | 1.232 (1.125, 1.348) | <0.001 |  | 1.502 (1.329, 1.698) | <0.001 |
|  |  |  |  |  |  |  |  |  |
| Lifetime complete pregnancy duration/per year | 1.026 (0.977, 1.078) | 0.311 |  | 1.049 (1.018, 1.081) | 0.002 |  | 1.059 (1.021, 1.099) | 0.002 |
| Lifetime incomplete pregnancy duration/per year | 1.361 (1.118, 1.658) | 0.002 |  | 1.067 (0.938, 1.213) | 0.325 |  | 1.418 (1.215, 1.655) | <0.001 |
| Gestation-to-reproductive lifespan duration ratio | 1.017 (1.004, 1.031) | 0.011 |  | 1.024 (1.015, 1.032) | <0.001 |  | 1.036 (1.026, 1.046) | <0.001 |
| Incomplete pregnancy-to-cumulative gestation duration ratio | 1.008 (1.004, 1.013) | <0.001 |  | 1.001 (0.998, 1.004) | 0.540 |  | 1.008 (1.004, 1.011) | <0.001 |

Abbreviations: CVD, cardiovascular disease.

^a^ Adjustments include age at enrollment, province, ethnicity, educational level, occupation, marital status, household annual income, tea drinking, coffee drinking, alcohol consumption, smoke status, physical activity in MET, age at menarche, OCP use, age at first born, history of ERT, and BMI.

^b^ Additionally adjusted diabetes in models.

**Supplemental Table 6.** **Sensitivity analyses among women without missing data on covariates (N=30,000).**

| Estrogen exposure indicators | diabetes ^a^ | |  | hypertension ^b^ | |  | CVD ^b^ | |
| --- | --- | --- | --- | --- | --- | --- | --- | --- |
|  | OR (95%CI) | *P* value |  | OR (95%CI) | *P* value |  | OR (95%CI) | *P* value |
| Reproductive lifespan/per year | 0.974 (0.963, 0.985) | <0.001 |  | 0.966 (0.960, 0.973) | <0.001 |  | 0.952 (0.944, 0.961) | <0.001 |
| Q1 (≤30y) | Ref. |  |  | Ref. |  |  | Ref. |  |
| Q2 (30.1-33.0y) | 0.868 (0.751, 1.003) | 0.055 |  | 0.935 (0.859, 1.018) | 0.121 |  | 0.802 (0.718, 0.896) | <0.001 |
| Q3 (33.1-36.0 y) | 0.861 (0.748, 0.992) | 0.038 |  | 0.780 (0.716, 0.850) | <0.001 |  | 0.655 (0.585, 0.735) | <0.001 |
| Q4 (>36.0y) | 0.738 (0.627, 0.869) | <0.001 |  | 0.637 (0.577, 0.704) | <0.001 |  | 0.551 (0.481, 0.631) | <0.001 |
| Endogenous estrogen exposure/per year | 0.981 (0.971, 0.990) | <0.001 |  | 0.970 (0.964, 0.976) | <0.001 |  | 0.957 (0.950, 0.963) | <0.001 |
| Q1 (≤24.25y) | Ref. |  |  | Ref. |  |  | Ref. |  |
| Q2(24.26-28.50y) | 0.923 (0.804, 1.059) | 0.253 |  | 0.815 (0.750, 0.886) | <0.001 |  | 0.756 (0.679, 0.841) | <0.001 |
| Q3(28.51-31.75y) | 0.797 (0.682, 0.932) | 0.004 |  | 0.749 (0.683, 0.822) | <0.001 |  | 0.607 (0.535, 0.689) | <0.001 |
| Q4 (>31.75y) | 0.682 (0.575, 0.808) | <0.001 |  | 0.620 (0.560, 0.686) | <0.001 |  | 0.439 (0.379, 0.509) | <0.001 |
| Lifetime cumulative gestation duration/per year | 1.053 (1.003, 1.105) | 0.039 |  | 1.059 (1.028, 1.092) | <0.001 |  | 1.081 (1.042, 1.122) | <0.001 |
| Q1 (≤1.50y) | Ref. |  |  | Ref. |  |  | Ref. |  |
| Q2(1.60-1.75y) | 1.379 (1.099, 1.729) | 0.005 |  | 1.111 (0.964, 1.280) | 0.147 |  | 1.077 (0.879, 1.320) | 0.472 |
| Q3(1.76-2.50y) | 1.268 (1.100, 1.460) | 0.001 |  | 1.088 (1.002, 1.182) | 0.044 |  | 1.328 (1.184, 1.491) | <0.001 |
| Q4(>2.50y) | 1.415 (1.208, 1.657) | <0.001 |  | 1.243 (1.133, 1.364) | <0.001 |  | 1.389 (1.223, 1.576) | <0.001 |
|  |  |  |  |  |  |  |  |  |
| Lifetime complete pregnancy duration/per year | 1.039 (0.988, 1.093) | 0.134 |  | 1.057 (1.025, 1.091) | <0.001 |  | 1.068 (1.028, 1.109) | 0.001 |
| Lifetime incomplete pregnancy duration/per year | 1.333 (1.085, 1.637) | 0.006 |  | 1.111 (0.974, 1.268) | 0.116 |  | 1.401 (1.194, 1.643) | <0.001 |
| Gestation-to-reproductive lifespan duration ratio | 1.023 (1.009, 1.037) | 0.001 |  | 1.028 (1.020, 1.037) | <0.001 |  | 1.039 (1.028, 1.049) | <0.001 |
| Incomplete pregnancy-to-cumulative gestation duration ratio | 1.008 (1.004, 1.013) | 0.004 |  | 1.277 (0.936, 1.742) | 0.122 |  | 1.007 (1.003, 1.011) | <0.001 |

Abbreviations: CVD, cardiovascular disease.

^a^ Adjustments include age at enrollment, province, ethnicity, educational level, occupation, marital status, household annual income, tea drinking, coffee drinking, alcohol consumption, smoke status, physical activity in MET, age at menarche, OCP use, age at first born, history of ERT, and BMI.

^b^ Additionally adjusted diabetes in models.

**Supplemental Table 7. Association between endogenous estrogen exposure and the risk of hypertension by using Possion regression with robust variance.**

| Estrogen exposure indicators | aRR (95%CI) | *P* value |
| --- | --- | --- |
| Reproductive lifespan/per year | 0.976 (0.972, 0.981) | <0.001 |
| Q1 (≤30y) | Ref. |  |
| Q2 (30.1-33.0y) | 0.954 (0.900, 1.011) | 0.112 |
| Q3 (33.1-36.0 y) | 0.841 (0.793, 0.891) | <0.001 |
| Q4 (>36.0y) | 0.733 (0.685, 0.786) | <0.001 |
| Endogenous estrogen exposure/per year | 0.980 (0.796, 0.984) | <0.001 |
| Q1 (≤24.25y) | Ref. |  |
| Q2(24.26-28.50y) | 0.876 (0.828, 0.927) | <0.001 |
| Q3(28.51-31.75y) | 0.825 (0.773, 0.880) | <0.001 |
| Q4 (>31.75y) | 0.722 (0.672, 0.776) | <0.001 |
| Lifetime cumulative gestation duration/per year | 1.030 (1.011, 1.050) | 0.002 |
| Q1 (≤1.50y) | Ref. |  |
| Q2(1.60-1.75y) | 1.093 (0.986, 1.212) | 0.092 |
| Q3(1.76-2.50y) | 1.086 (1.021, 1.155) | 0.009 |
| Q4(>2.50y) | 1.165 (1.090, 1.245) | <0.001 |
|  |  |  |
| Lifetime complete pregnancy duration/per year | 1.028 (1.008, 1.048) | 0.005 |
| Lifetime incomplete pregnancy duration/per year | 1.094 (1.004, 1.192) | 0.040 |
| Gestation-to-reproductive lifespan duration ratio | 1.016 (1.011, 1.021) | <0.001 |
| Incomplete pregnancy-to-cumulative gestation duration ratio | 1.002 (1.065, 1.073) | 0.038 |

Abbreviations aRR, adjusted relative ratio; CI, confidence interval

Adjustments include age at enrollment, province, ethnicity, educational level, occupation, marital status, household annual income, tea drinking, coffee drinking, alcohol consumption, smoke status, physical activity in MET, age at menarche, OCP use, age at first born, history of ERT, and BMI.

**Supplemental Table 8.** **E-values of associations between estrogen exposure indicators and the risk of cardiovascular disease.**

|  | Diabetes / E value (CI) | Hypertension / E-value (CI) | CVD / E-value (CI) |
| --- | --- | --- | --- |
| Reproductive lifespan/per year | 1.205 (1.155) | 1.214 (1.188) | 1.273 (1.242) |
| Q1 (≤30y) | Ref. | Ref. | Ref. |
| Q2 (30.1-33.0y) | 1.574 (1.084) | 1.310 (1.000) | 1.698 (1.394) |
| Q3 (33.1-36.0 y) | 1.612 (1.677) | 1.853 (1.618) | 2.444 (2.111) |
| Q4 (>36.0y) | 2.049 (1.601) | 2.394 (2.103) | 3.017 (2.574) |
| Endogenous estrogen exposure/per year | 1.174 (1.129) | 1.209 (1.183) | 1.265 (1.238) |
| Q1 (≤24.25y) | Ref. | Ref. | Ref. |
| Q2(24.26-28.50y) | 1.476 (1.000) | 1.805 (1.574) | 1.972 (1.684) |
| Q3(28.51-31.75y) | 1.842 (1.412) | 1.968 (1.712) | 2.835 (2.444) |
| Q4 (>31.75y) | 2.369 (1.883) | 2.646 (2.336) | 4.048 (3.464) |
| Lifetime cumulative gestation duration/per year | 1.233 (1.000) | 1.276 (1.163) | 1.344 (1.084) |
| Q1 (≤1.50y) | Ref. | Ref. | Ref. |
| Q2(1.60-1.75y) | 1.886 (1.221) | 1.524 (1.000) | 1.714 (1.084) |
| Q3(1.76-2.50y) | 1.783 (1.389) | 1.409 (1.116) | 2.148 (1.834) |
| Q4(>2.50y) | 2.019 (1.583) | 1.721 (1.465) | 2.295 (1.936) |
|  |  |  |  |
| Lifetime complete pregnancy duration/per year | 1.163 (1.000) | 1.255 (1.133) | 1.279 (1.133) |
| Lifetime incomplete pregnancy duration/per year | 2.091 (1.542) | 1.406 (1.000) | 1.588 (1.000) |
| Gestation-to-reproductive lifespan duration ratio | 1.158 (1.091) | 1.181 (1.143) | 1.229 (1.189) |
| Incomplete pregnancy-to-cumulative gestation duration ratio | 1.098 (1.067) | 1.047 (1.000) | 1.047 (1.000) |

Abbreviations: CVD, cardiovascular disease.

**Supplemental Figure 1. Restricted cubic spline plots for the association between endogenous estrogen exposure and the risk of diabetes, hypertension, and CVD.**


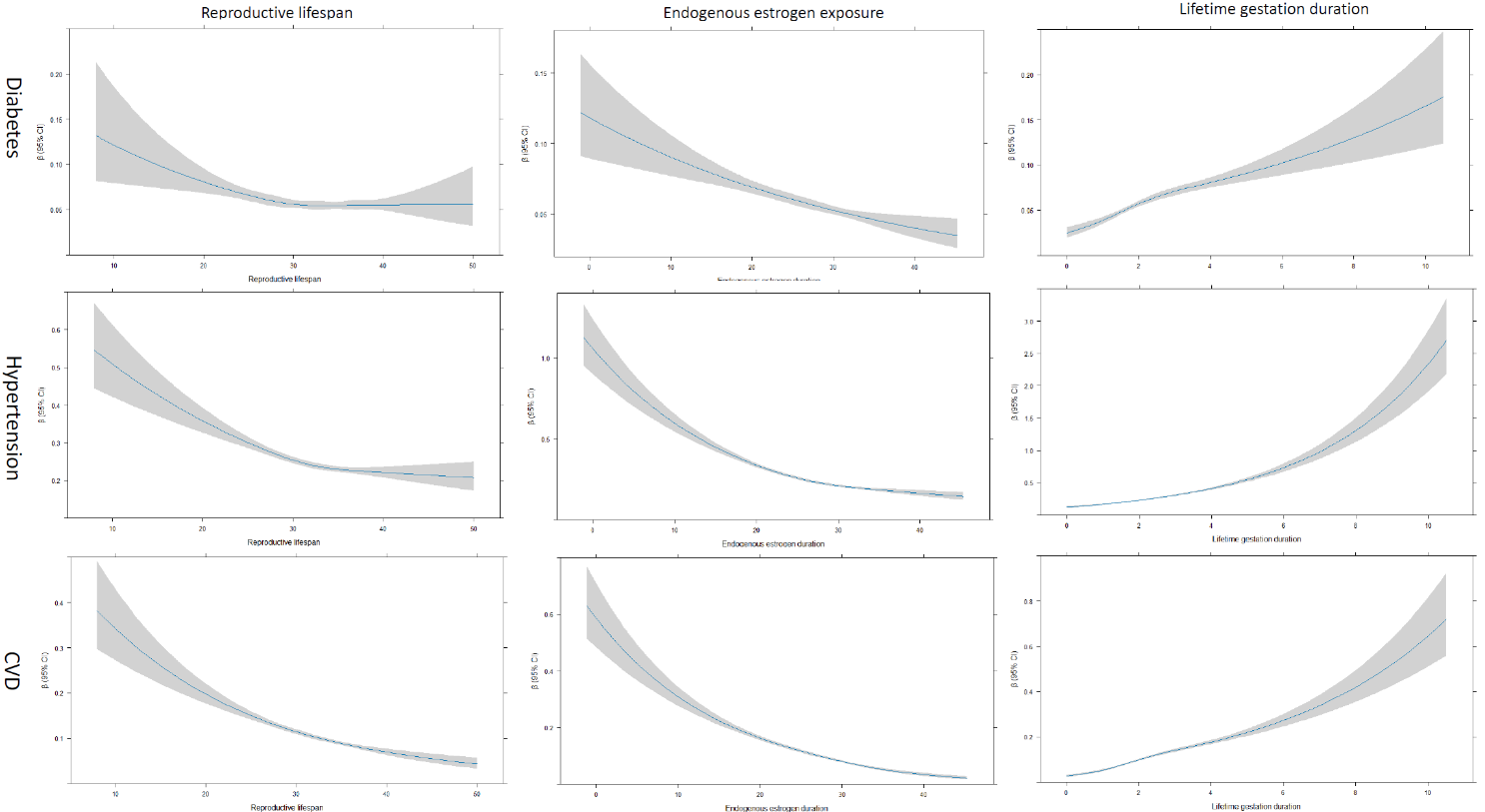

Supplement: Supplementary file 1 — Supplementary Material 1 [file 12905_2025_4030_MOESM1_ESM.docx]
